# Editorial: Endemic Plants: Experimental and Theoretical Insights Into Properties of Bioactive Metabolites With Therapeutic Potential

**DOI:** 10.3389/fchem.2021.786865

**Published:** 2021-10-25

**Authors:** Carolina Otero, Jorge Ignacio Martínez-Araya, Jorge M. del Campo, Felipe Gordillo-Fuenzalida

**Affiliations:** ^1^ School of Chemistry and Pharmacy, Faculty of Medicine, Universidad Andres Bello, Santiago, Chile; ^2^ Departamento de Ciencias Químicas, Facultad de Ciencias Exactas, Universidad Andrés Bello, Santiago, Chile; ^3^ Departamento de Física y Química Teórica, Facultad de Química, Universidad Nacional Autónoma de México, Ciudad de México, Mexico City, Mexico; ^4^ Laboratorio de Microbiología Aplicada, Centro de Biotecnología de los Recursos Naturales, Facultad de Agronomía y Ciencias Forestales, Universidad Católica del Maule, Talca, Chile

**Keywords:** *in silico*, endemic plants, secondary metabolites, anti-inflammatory, anti-oxidant

## Introduction

Plants are currently used in alternative and ancestral medicine and correspond to the best resource in drug discovery known so far. It has primarily been reported that infusion of herbs as aromatic drinks or tea can protect and heal our organism. From the scientific point of view, it is known that water-soluble substances (normally secondary metabolism) have beneficial effects on people’s health. Researchers have taken advantage of plant metabolites as a fundamental part of the healing process, consuming them to treat diseases or relieve pathological conditions. Then, once consumed, these molecules can interact with different targets (including cell membranes), and in some cases, they can even be internalized by cells triggering intracellular responses. The continued search for potential compounds against therapeutic targets is still necessary. Fast screening *in silico* using bioinformatics tools has allowed finding new metabolite candidates derived from natural resources.

**GRAPHICAL ABSTRACT:**
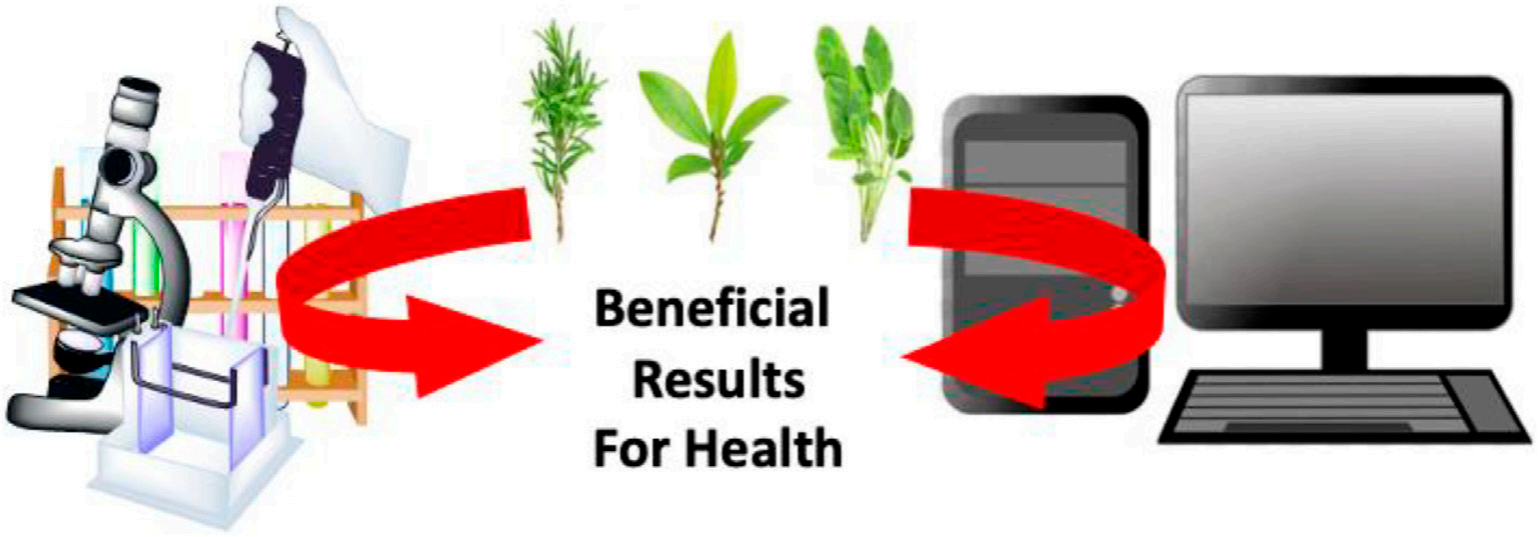


## Computational Tools

Informatics’s role in biomedical science has become not a supplementary tool but a necessary support nowadays since it allows researchers to optimize their work in search of new molecules or new properties of well-known molecules. The maturity attained by Quantum Chemistry, Molecular Dynamics, and Molecular Mechanics provides helpful guidance for activities performed by experimentalist scientists, providing support to their scientific investigation that concerns the health arena. The present Research Topic exposes some examples of this theoretical and experimental combined work to reveal relevant properties that secondary metabolites might present.

## Conclusion of This Topic

Overall, this topic covers contributions that range from analytical chemistry to computational chemistry, applied to endemic Brazilian plants such as *Byrsonima intermedia* and *Serjania marginata* found to possess antiseptic, antimicrobial, anti-haemorrhagic, cicatrizant, and anti-inflammatory properties, computational peptidology applied to determination of the chemical reactivity and bioactivity properties of plant cyclopeptides isolated from *Rosaceae*, and antioxidant *in silico* prediction of moracin-C and iso-moracin-C isomers against the OOH free radical. *Annona muricata*, a plant that has been reported to demonstrate significant antiviral properties against the human immunodeficiency virus, herpes simplex virus, human papilloma virus, hepatitis C virus and dengue virus, to *in silico* search of GSK3-β inhibitors as a potential Alzheimer’s disease treatment.

## Future Prospects

Thanks to the studies shown in this section, we hope that more researchers will join the search for new properties of biomolecules extracted from different parts of endemic plants. In this sense, Latin America, particularly South America, has enormous potential due to its rich biodeversity that invites scientists to continue the search of these biomolecules with biomedical and biotechnological application.

